# A Case of Cutaneous Botryomycosis of the Lower Leg in a Young Adult Male

**DOI:** 10.7759/cureus.16502

**Published:** 2021-07-20

**Authors:** Fadie Aziz, Ferdinand Ong, Roneil N Parikh, Auerilius E Hamilton

**Affiliations:** 1 General Surgery, Campbelltown Hospital, Sydney, AUS; 2 Colorectal Surgery, St. George Hospital, Sydney, AUS; 3 Colorectal Surgery, Royal Prince Alfred Hospital, Sydney, AUS

**Keywords:** botryomycosis, skin graft

## Abstract

We present a case of cutaneous botryomycosis of the lower leg in a young adult male. Botryomycosis is a chronic granulomatous response to bacterial infection. As a cutaneous lesion, it can easily be mistaken for a malignant, autoimmune or inflammatory mass. We were able to successfully treat our patient with primary surgical excision, vacuum-assisted closure (V.A.C.™) dressing and subsequent split thickness skin graft (STSG). Use of a V.A.C. dressing with subsequent grafting has not previously been reported in the literature.

## Introduction

We report a case of cutaneous botryomycosis of the leg in a 37-year-old Caucasian male treated with primary surgical excision, vacuum-assisted closure ([V.A.C.™], KCI-Acelity, San Antonio, TX, USA) and subsequent split-thickness skin grafting (STSG). Botryomycosis is a disease of chronic inflammatory lesions caused by a granulomatous response to bacterial infection [1]. The lesions are often hard and woody, show mixed areas of necrosis, inflammation and suppuration macroscopically and typically elicit a surrounding inflammatory response. The appearance makes it easily mistakable for other malignant, autoimmune or inflammatory lesions, especially since it is not often encountered. Diagnosis may not always be possible with biopsy. If left untreated, these lesions can behave like malignancies, invading into deeper tissues, forming abscesses and having the potential to form fistulas. Features that should alert one to a diagnosis of botryomycosis include rapid growth, response to antibiotics and positive culture on biopsy or swab.

With only 200 reported cases in the English literature, botryomycosis is a rare entity. There are no previously reported cases of surgical excision with subsequent use of a V.A.C. dressing and skin grafting and we believe the case presented below shows that this is a suitable reconstructive option. 

## Case presentation

**Figure 1:**
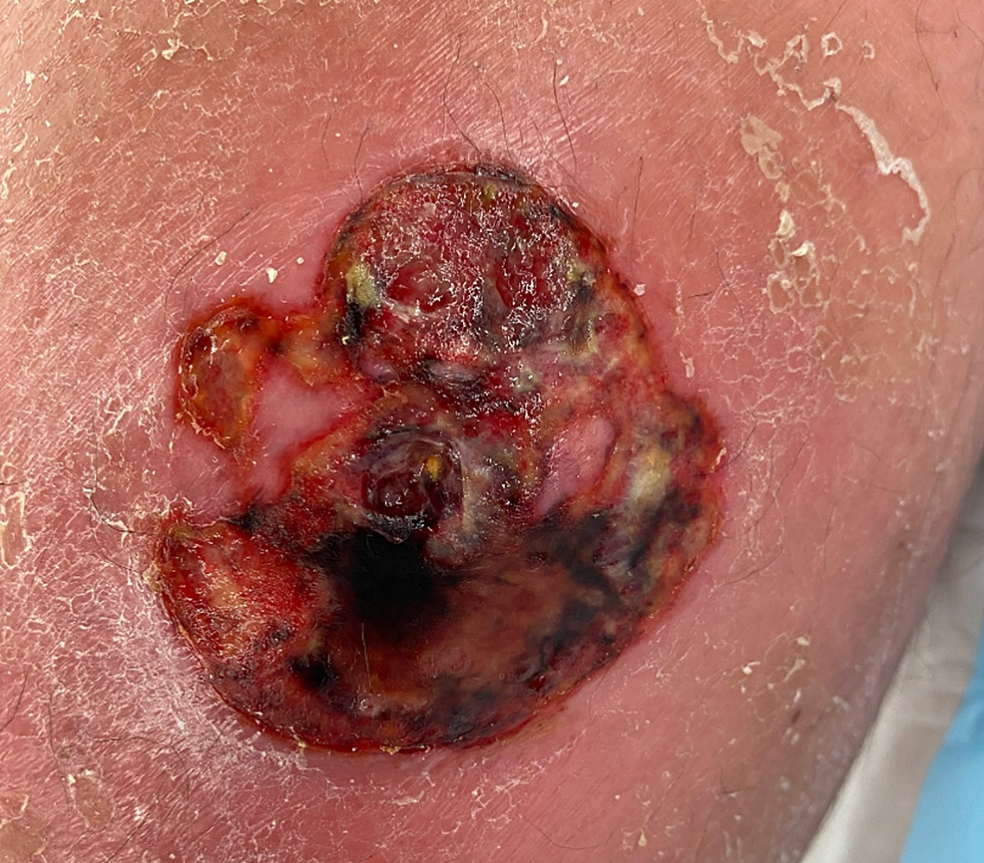
Pre-operative photograph of right medial calf lesion showing areas of ulceration and necrosis

Our patient was a 37-year-old Caucasian male who presented to the emergency department with a 30-day history of a necrotic, ulcerating right calf lesion. He was systemically well and did not have any pain. He denied any preceding trauma or inciting triggers. He had been treated by his general practitioner with oral amoxicillin/clavulanic acid, however, the lesion failed to improve. His past medical history included alcoholism, anxiety disorder, recurrent lower limb cellulitis and a right ankle fracture requiring internal fixation. He was an active smoker with no history of diabetes mellitus. On examination, the 5cm diameter lesion had areas of ulceration and necrosis with surrounding cellulitis (Figure 1). There was no clinical evidence of peripheral vascular disease or neuropathy. His white cell count was 11.4x109/L and his C-reactive protein was 6.3mg/L. An x-ray of his right leg did not show evidence of osteomyelitis. Wound culture grew both methicillin-resistant *Staphylococcus aureus *(MRSA) and *Enterococcus*. 

The patient underwent primary surgical excision of the lesion. Macroscopically, there was low-grade inflammation which is why we delayed grafting. A V.A.C. was applied with negative pressure at 125mmHg with continuous suction. Histopathology with Periodic acid-Schiff staining revealed extensive suppurative necrosis with fibrosis. The base of the lesion contained granules with blue-stained bacterial organisms surrounded by an intense eosinophilic coat. This finding is in keeping with “Splendore-Hoeppli Phenomenon”, a finding characteristic of botryomycosis. There was no evidence of malignancy and no fungal organisms were identified. He was treated with 48 hours of intra-venous clindamycin and discharged with a two-week course of oral clindamycin as guided by the infectious diseases team. The V.A.C. was managed by community nurses. 

**Figure 2:**
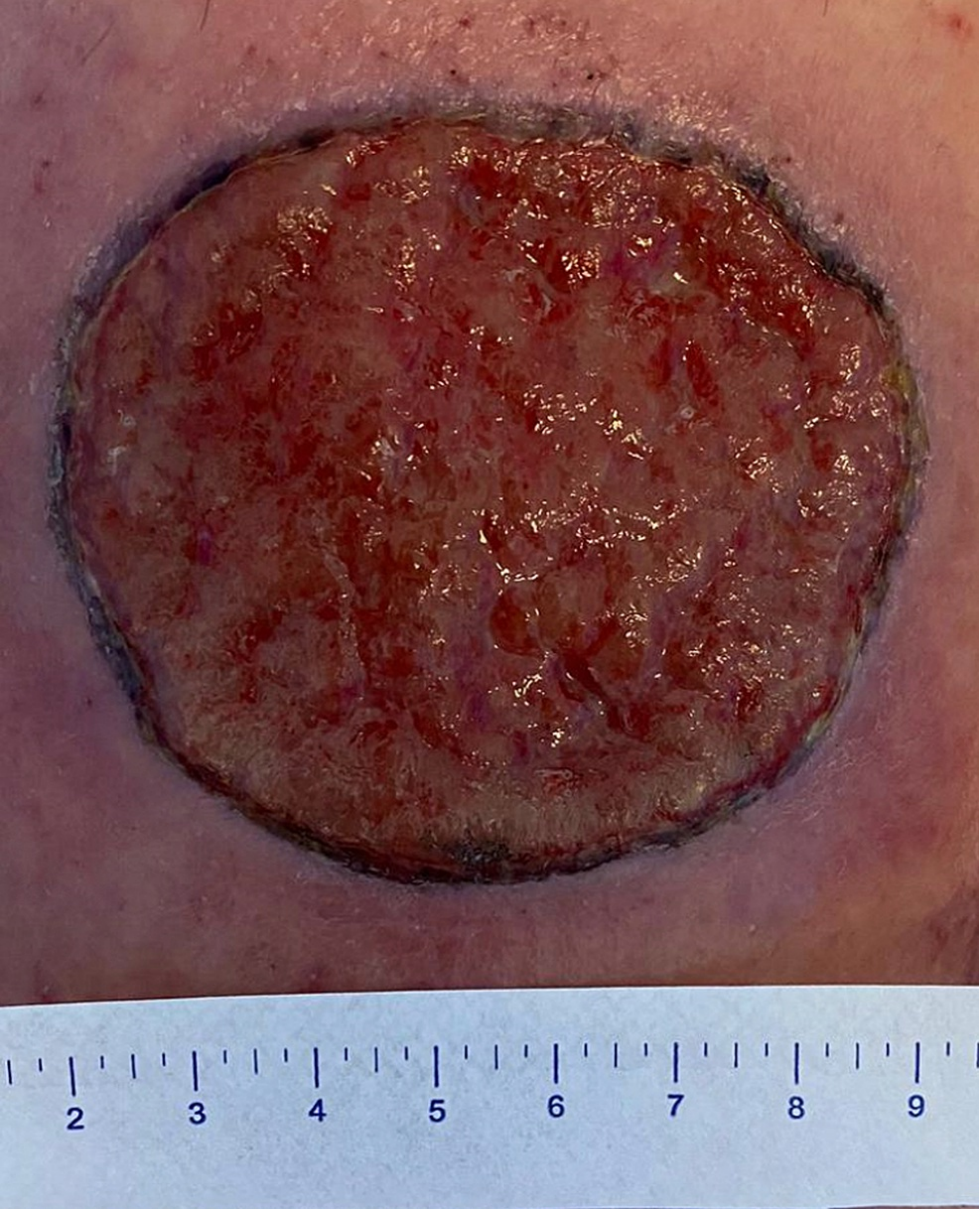
Wound bed on day 30 post excision

Upon review in the clinic, the wound bed appeared healthy with good granulation tissue. The cellulitis had improved (Figure 2). 

**Figure 3:**
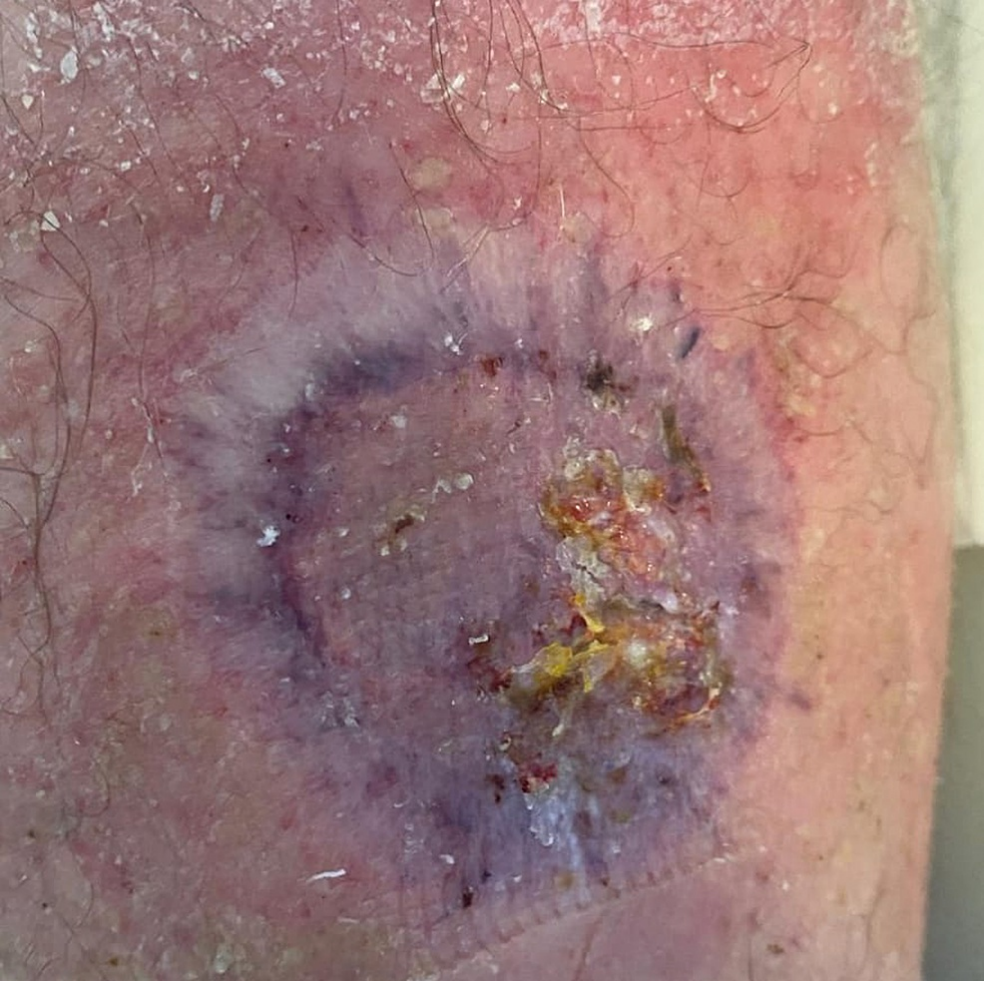
Grafted wound

The patient’s V.A.C. dressing was only attended to in clinic due to anxiety surrounding changing these dressings at home. Once the inflammation settled and the wound base appeared healthy, the patient was re-admitted and underwent a split-thickness skin grafting procedure. Upon follow-up, the graft took well (Figure 3).

## Discussion

Botryomycosis may be either cutaneous (75% of cases) or visceral, typically affecting the lungs but possibly involving the liver, spleen, brain or kidneys [2]. Its name derives from the Greek “botryose” meaning cluster of grapes and “mycosis” referring to the original perception that it was caused by fungi. This name refers to the characteristic histopathological finding of granules containing bacteria surrounded by an eosinophilic zone; the so-called “Splendore-Hoeppli Phenomenon” (SHP). Whilst characteristic, this finding is not pathognomonic and may be seen in actinomycosis as well as fungal and helminthic infection [3].

The most frequently implicated organism is *Staphylococcus aureus* (40% of cases) followed by *Pseudomonas aeruginosa* (20% of cases) and *Escherichia coli*. Other implicated organisms include *Proteus* and *Streptococcus* species [2]. Cutaneous botryomycosis has an insidious onset and is thought to arise from an inoculating trauma and therefore is usually found to affect the head, neck, and extremities. It can manifest as nodules, non-healing ulcers, sinuses or fistulae and has the potential to invade deeper. Predisposing factors include alcoholism, diabetes mellitus, immunosuppression, and malnutrition [4].

Botryomycosis is thought to arise when there is a balance between the virulence of the involved bacteria, the size of inoculum and an impaired host response. Too little inoculum, low bacterial virulence and a robust host response favours clearance of the infection. In the opposite scenario, bacterial infection overwhelms the host response forming an abscess [5]. Microscopically, this balance manifests as the SHP, whereby antigen-antibody complexes and fibrin form an eosinophilic coat that surrounds bacterial granules and prevents phagocytosis [6].

There are reported cases of successful management with antibiotics exclusively, however combination with surgical debridement is often used [7,8]. In our case, we elected for antibiotic therapy with surgical excision and subsequent STSG given the size and location of the lesion and the presence of macroscopic necrosis. Use of an STSG has been reported twice previously in the literature with one of the grafts failing [4,9]. Use of a V.A.C. dressing with subsequent STSG once the infection resolved has not been previously published. 

## Conclusions

In conclusion, this rare clinical entity may easily be mistaken for a malignant lesion, or a non-infective inflammatory or autoimmune mass. In cases where lesions are smaller and not yet necrotic, primary antibiotic therapy would be feasible. However, larger lesions will most likely be non-responsive due to a lack of antibiotic penetrance and the presence of necrotic tissue requiring debridement. Our case highlights that surgical excision and V.A.C. dressing allow time to treat the infection. An STSG is a suitable reconstructive option.
